# On the Representability of Complete Genomes by Multiple Competing Finite-Context (Markov) Models

**DOI:** 10.1371/journal.pone.0021588

**Published:** 2011-06-30

**Authors:** Armando J. Pinho, Paulo J. S. G. Ferreira, António J. R. Neves, Carlos A. C. Bastos

**Affiliations:** Signal Processing Lab, IEETA/DETI, University of Aveiro, Aveiro, Portugal; The Centre for Research and Technology Hellas, Greece

## Abstract

A finite-context (Markov) model of order 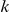 yields the probability distribution of the next symbol in a sequence of symbols, given the recent past up to depth 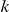. Markov modeling has long been applied to DNA sequences, for example to find gene-coding regions. With the first studies came the discovery that DNA sequences are non-stationary: distinct regions require distinct model orders. Since then, Markov and hidden Markov models have been extensively used to describe the gene structure of prokaryotes and eukaryotes. However, to our knowledge, a comprehensive study about the potential of Markov models to describe complete genomes is still lacking. We address this gap in this paper. Our approach relies on (i) multiple competing Markov models of different orders (ii) careful programming techniques that allow orders as large as sixteen (iii) adequate inverted repeat handling (iv) probability estimates suited to the wide range of context depths used. To measure how well a model fits the data at a particular position in the sequence we use the negative logarithm of the probability estimate at that position. The measure yields information profiles of the sequence, which are of independent interest. The average over the entire sequence, which amounts to the average number of bits per base needed to describe the sequence, is used as a global performance measure. Our main conclusion is that, from the probabilistic or information theoretic point of view and according to this performance measure, multiple competing Markov models explain entire genomes almost as well or even better than state-of-the-art DNA compression methods, such as XM, which rely on very different statistical models. This is surprising, because Markov models are local (short-range), contrasting with the statistical models underlying other methods, where the extensive data repetitions in DNA sequences is explored, and therefore have a non-local character.

## Introduction

Since the work of Grumbach and Tahi [1], many contributions have been made in the area of DNA data compression (see, for example, [2]–[10] and for a recent review [11]). These works explore the non-stationary nature of DNA sequence data, which are characterized by an alternation between regions of relatively high and low entropy. Typically, there are two compression approaches, one based on Lempel-Ziv-like substitutional procedures [12] (that usually perform well on repetitive, low entropy regions) and another based on low-order context-based (Markov) arithmetic coding (better suited for regions of high entropy).

According to the substitutional paradigm, repeated regions of the DNA sequence are represented by a pointer to a past occurrence of the repetition and by the length of the repeating sequence. Both exact and approximate repetitions have been explored, as well as their inverted complements.

Markov modeling has long been applied to DNA data sequences (see, for example, the works of Borodovsky *et al.* [13], [14] and of Tavaré and Song [15]). Since then, a large number of publications have addressed this topic, although mainly with the aim of proposing techniques for gene finding (some examples can be found in [16]–[22]). Other applications, such as the detection of short inverted DNA segments [23], the assessment of the statistical significance of DNA patterns [24] or the identification of CpG islands [25], have also relied on Markov models. However, Markov models have never been used as the sole paradigm for DNA sequence modeling or compression. In this paper, we address a modeling question that we do believe has not been satisfactorily answered before: How well can **complete genomes** be described using exclusively a combination of Markov models? We seek descriptions that are good in the sense of the minimal description length principle [26], i.e., that require as few bits as possible for representing the information.

To investigate this matter, we developed a method based on multiple competing finite-context models that incorporate features found in DNA sequence data, such as the existence of inverted repeats. Finite-context models are computational models that provide a probability estimate of the next DNA base, given the recent past of the sequence, in accordance with the Markov property.

There is a close connection between compression and modeling. Compression methods depend on statistical models of the data. If a compression method outperforms another, it is because the underlying statistical model is better suited to the data. Conversely, if a statistical data model explains a string of data very well, that is, if it provides good estimates of the distribution of each data symbol, then it is conceivable that its application in sequence compression might lead to good compression performance.

To measure how well a model fits the data at a particular position in the sequence we use the negative logarithm of the probability estimate at that position. The measure yields information profiles of the sequence, which are of independent interest. The average over the entire sequence, which amounts to the average number of bits per base needed to describe the sequence, is used as a global performance measure.

Our experimental results show that the ability of multiple competing finite-context models to describe DNA sequences is surprisingly close to that attained by more complex state-of-the-art DNA compression methods, such as XM [10]. In fact, for small-sized sequences, the finite-context models perform better.

XM, the method that we use as the reference to compare the performance of the finite-context models, relies on a mixture of experts for providing symbol by symbol probability estimates, which are then used for driving an arithmetic encoder. The algorithm comprises three types of experts: (1) order-2 Markov models; (2) order-1 context Markov models, i.e., Markov models that use statistical information only of a recent past (typically, the 512 previous symbols); (3) the copy experts, that consider the next symbol as part of a copied region from a particular offset. The probability estimates provided by the set of experts are then combined using Bayesian averaging and sent to the arithmetic encoder.

Besides a global comparison, based on the average of the negative logarithm of the probability estimates (i.e., the average of the per base information content) performed for several genomes of various sizes, we also provide some samples of the local profiles of the so-called information sequences [27]. These information sequences contain the per base information content generated by the models (measured in bits), allowing, for example, the comparative analysis of long DNA sequences [28], the classification of biological sequences [29] or sequence alignment [30]. In addition, we show an example of the context depth profile produced along the sequences, that might have independent interest.

As we mentioned before, in this paper we explore multiple competing finite-context models, with the aim of finding how well complete DNA data sequences can be described exclusively by this modeling paradigm.

As far as we know, this paper provides the first comprehensive investigation of the extent to which Markov models explain DNA data. We believe that this is important because it provides evidence that complete DNA data sequences can be reasonably well described by statistical models that rely only on the immediate past of the sequence. In other words, local, short-range models perform as well as or better than non-local models built in the state-of-the-art compression methods. Since the search for better data compression methods is intimately related to the problem of finding better data models, this work contributes to an improved understanding of the laws that govern the DNA data, an objective that has been long pursued (see, for example, [1], [31]–[33]).

## Materials and Methods

### DNA data sequences

In this study, we used the complete DNA sequences of eleven species of various sizes. The genomes were obtained from the following sources:

- *Homo sapiens*, Build 33, from the National Center for Biotechnology Information (NCBI) (ftp://ftp.ncbi.nlm.nih.gov/genomes/H_sapiens/April_14_2003);
- *Arabidopsis thaliana*, TAIR 9, from The Arabidopsis Information Resource (ftp://ftp.arabidopsis.org/home/tair/Sequences/whole_chromosomes);
- *Candida albicans*, Assembly 21, from the Candida Genome Database (http://www.candidagenome.org/download/sequence/Assembly21);
- *Staphylococcus aureus aureus* MSSA476, NC002953, from the NCBI (ftp://ftp.ncbi.nlm.nih.gov/genomes/Bacteria/Staphylococcus_aureus_aureus_MSSA476);
- *Thermococcus kodakarensis* KOD1, NC006624, from the NCBI (ftp://ftp.ncbi.nlm.nih.gov/genomes/Bacteria/Thermococcus_kodakaraensis_KOD1);
- *Methanocaldococcus jannaschii* DSM 2661, NC000909, from the NCBI (ftp://ftp.ncbi.nlm.nih.gov/genomes/Bacteria/Methanococcus_jannaschii);
- *Schizosaccharomyces pombe*, NC001326, NC003421, NC003423, NC003424, from the NCBI (ftp://ftp.ncbi.nlm.nih.gov/genomes/Fungi/Schizosaccharomyces_pombe);
- *Mycoplasma genitalium*, NC000908, from the NCBI (ftp://ftp.ncbi.nlm.nih.gov/genomes/Bacteria/Mycoplasma_genitalium);
- *Aspergillus nidulans* FGSC A4 uid13961, from the NCBI (ftp://ftp.ncbi.nlm.nih.gov/genomes/Fungi/Aspergillus_nidulans_FGSC_A4_uid13961/);
- *Escherichia coli* K12 MG1655, NC000913, from the NCBI (ftp://ftp.ncbi.nlm.nih.gov/genomes/Bacteria/Escherichia_coli_K_12_substr__MG1655_uid57779/);
- *Saccharomyces cerevisiae*, from the NCBI (ftp://ftp.ncbi.nlm.nih.gov/genomes/Saccharomyces_cerevisiae/).

### Finite-context models

Consider an information source that generates symbols, 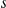, from a finite alphabet 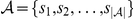, where 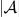 denotes the size of the alphabet. In the case of DNA data, 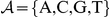 and, therefore, 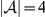. Also, consider that the information source has already generated the sequence of 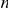 symbols 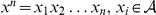. A finite-context model assigns probability estimates to the symbols of the alphabet, regarding the next outcome of the information source, according to a conditioning context computed over a finite and fixed number, 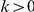, of the most recent past outcomes 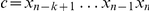 (order-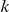 finite-context model) [34]–[36]. The number of conditioning states of the model is 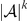, determining the model complexity or cost. The context, 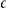, varies along the sequence, i.e., it depends on the position 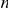. However, for alleviating the notation, we considered this dependency implicit and, therefore, when we refer to 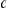 we mean the value of the context at the location that should be easily inferred from the formula where it occurs.

The probability estimates, 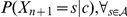, are usually calculated using symbol counts that are accumulated while the sequence is processed, which makes them dependent not only of the past 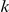 symbols, but also of 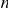. In other words, these probability estimates are generally time varying.

**Figure 1:**
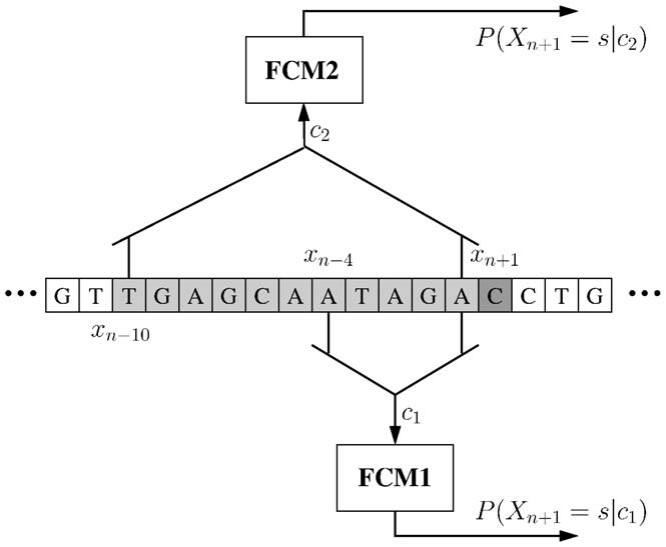
Example of finite-context models. In this example, 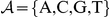 and the context depths, 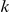, are 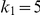 and 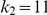. The probability of the next outcome, 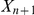, is conditioned by the last 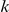 outcomes. When more than one model is running competitively, the particular context depth used is chosen on a block basis.

Table 1 shows an example of how statistical data are usually collected in finite-context modeling. In this example, an order-5 finite-context model is presented (as that of the FCM1 model of Fig. 1). Each row represents a probability model that is used to represent a given symbol according to the last processed symbols (five in this example). The counters are updated each time a symbol is processed.

**Table 1:** Probability models.

Context, 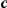	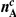	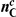	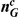	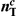	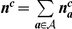
AAAAA	23	41	3	12	79
					
ATAGA	16	6	21	15	58
					
GTCTA	19	30	0	4	53
					
TTTTT	8	2	18	11	39

Simple example illustrating how statistical data are typically collected in finite-context models. Each row of the table represents a probability model at a given instant 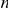. In this example, the particular model that is chosen for encoding a symbol depends on the last five processed symbols (order-5 context).

The theoretical per symbol information content average provided by the finite-context model after having processed 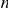 symbols is given by
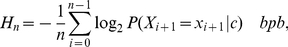
(1)where “bpb” stands for “bits per base”. Recall that the entropy of any sequence of four symbols is limited to two bits per symbol, a value that is obtained when the symbols are independent and equally likely, and that the fewer the number of bits produced the better is the model.

One of the drawbacks of implementing the finite-context models using the approach illustrated in Table 1 is that the memory requirements grow exponentially with 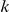. In fact, the total number of counters needed in this case is 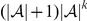. For DNA data, and even considering only two-byte counters, this would imply about 40 Gbytes of memory for implementing an order-16 model. However, this table would also be very sparse, because the maximum number of different words of size 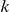 that can be found in a sequence of length 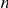 is clearly upper bounded by 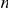. Using this simple observation and appropriate data structures such as hash-tables, we managed to implement a computer program that allows using finite-context models of orders up to sixteen in a laptop computer with 3 Gbytes of memory (the source code of this computer program is publicly available in ftp://www.ieeta.pt/~ap/codecs/DNAEnc3.tar.gz).

### Updating the inverted complements

Frequently, DNA sequences contain sub-sequences that are reversed and complemented copies of some other sub-sequences. These sub-sequences are named “inverted repeats”. As mentioned before, this particularity of DNA sequence data is used by most of the DNA compression methods that have been proposed and that rely on the sliding window searching paradigm.

For exploring the inverted repeats of a DNA sequence, besides updating the corresponding counter after encoding a symbol, we also update another counter that we determine in the following way [37]. Consider the example given in Fig. 1 (FCM1 model), where the context is the string “ATAGA” and the symbol to encode is “C”. Reversing the string obtained by concatenating the context string and the symbol, i.e., “ATAGAC”, we obtain the string “CAGATA”. Complementing this string (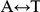, 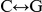), we get “GTCTAT”. Now we consider the prefix “GTCTA” as the context and the suffix “T” as the symbol that determines which counter should be updated. Therefore, according to this procedure, we take into consideration the inverted repeats if, after encoding symbol “C” of the example FCM1 of Fig. 1, the counters are updated according to Table 2. As shown in [37], this provides additional modeling performance.

**Table 2:** Updating the inverted repeats.

Context, 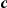	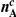	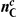	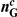	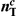	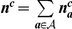
AAAAA	23	41	3	12	79
					
ATAGA	16	**7**	21	15	59
					
GTCTA	19	30	10	**5**	54
					
TTTTT	8	2	18	11	39

Table 1 updated after processing symbol “C” according to context “ATAGA” (see example of Fig. 1) and taking the inverted repeats property into account.

### Multiple competing models

DNA sequence data are non-stationary. In fact, one of the reasons why most DNA compression algorithms use a mixture of two methods, one based on repetitions and the other relying on low-order finite-context models, is to try to cope with the non-stationary nature of the data. We also follow this line of reasoning, i.e., that of using different models along the sequence. However, unlike the other approaches, we use exclusively the finite-context paradigm for modeling the data, changing only the order of the model as the characteristics of the data change. More precisely, we explore an approach based on multiple finite-context models of different orders that compete for encoding the data.

Using several models with different orders allows a better handling of DNA regions with diverse characteristics. Therefore, although these multiple models are continuously updated, only the best one is used for encoding a given region. For convenience, the DNA sequence is partitioned into non-overlapping blocks of fixed size, which are then encoded by one (the best one) of the finite-context models. Figure 1 shows an example where two competing finite-context models are used. In this example, each model collects statistical information from a context of depth 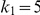 and 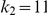, respectively. At time 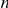, the two conditioning contexts are 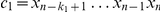 and 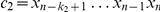.

### Estimating the probabilities

How to estimate probabilities based on counting the occurrences of past events has been a problem addressed by several researchers, going back at least to the works of Bayes and Laplace [38], [39].

The central problem is the estimation of the probability of events that have never been observed (this is also known as the pseudocount estimation problem). For that purpose, we use an estimator that is a generalization of earlier formulae (see, for example, [40]–[45]), which is given by
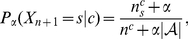
(2)where 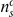 represents the number of times that, in the past, the information source generated symbol 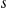 having 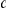 as the conditioning context and where
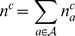
(3)is the total number of events that has occurred so far in association with context 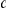. It is important to note that defining
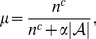
(4)the estimator can be rewritten as
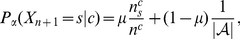
(5)revealing a linear interpolation between the maximum likelihood estimator and the uniform distribution. This also shows that when the total number of events, 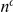, is large, the estimator behaves as a maximum likelihood estimator (when 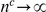, 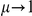), regardless of the value of 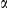. Therefore, the main interest in the estimator of (2) is when 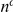 is small, in which case the value of 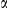 plays a key role. Moreover, it can also be seen that the parameter 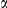 controls the probability assigned to previously unseen (but possible) events, i.e., the probability when 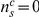. This probability is given by
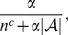
(6)which decreases faster with 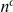 for smaller values of 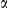.

The estimator described in (2) assumes a Dirichlet prior, 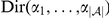, over the probabilities that are being estimated, with 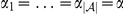. Dirichlet mixtures have also been used, for example in the context of protein family modeling (see, for instance, [46], [47]).

## Results and Discussion

Each organism under analysis was processed using eight competing finite-context models with context depths 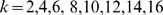. The decision of which depth to use was taken on a block by block basis, using blocks of two hundred DNA bases. This block size, although not optimal for every sequence, has revealed to be on average a good compromise.

Since we are interested in evaluating the performance of the models, we used the average number of bits per DNA base (bpb) provided by these models, as a measure of their fitness to the data. This is essentially the value provided by (1) when 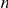 equals the length of the sequence, i.e., after processing the whole sequence. Besides this per symbol average information content, the overhead required to indicate the depth of the particular finite-context model used in each data block was also considered. Note that, for blocks of two hundred bases, and without further modeling, this implies a small overhead of 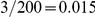 bpb (recall that the eight possible context depths can be represented with three bits). Nevertheless, we also used a finite-context model for representing this information in a more efficient way. It was found, experimentally, that an order-4 model was able to provide a good performance.

For comparison, we processed the DNA sequences using the single finite-context model approach. In this case, the best context depth was used. For genomes composed of several chromosomes, the best context depth was determined for each chromosome. The results regarding this approach are presented in the “FCM-S” column of Table 3, whereas the results obtained with the multiple competing models are shown in column “FCM-M”. We used the currently best-performing DNA compression algorithm, XM [10], for evaluating the overall performance of the multiple competing finite-context models in comparison with the state-of-the-art technique for DNA sequence compression. Also, with the aim of providing an additional term of comparison, we include the results attained by another DNA compression method, developd by Manzini *et al.* [6], because it is a fast, although competitive DNA compressor. This technique is based on fingerprints for fast pattern matching, and relies on fallback mechanisms for encoding the regions where matching fails, which are order-2 (*DNA2*) or order-3 (*DNA3*) finite-context models. The results presented in Table 3 correspond to the average number of bits actually generated.

**Table 3:** Results for eleven complete genomes.

Organism	Size	DNA3	FCM-S	FCM-M		XM50		XM200	
	Mb	bpb	bpb	bpb	secs	bpb	secs	bpb	secs
*H. sapiens*	2832.18	1.779	1.773	1.695	22529	1.644	92461	1.618	129374
*A. thaliana*	119.48	1.836	1.911	1.821	1106	1.736	1614	1.730	3423
*A. nidulans*	29.54	1.977	1.987	1.978	177	1.968	143	1.968	146
*C. albicans*	14.32	1.872	1.882	1.864	93	1.861	119	1.861	146
*S. pombe*	12.59	1.886	1.926	1.887	75	1.865	97	1.865	140
*S. cerevisiae*	12.16	1.906	1.940	1.906	77	1.892	50	1.892	51
*E. coli*	4.64	1.915	1.937	1.901	27	1.914	39	1.914	50
*S. aureus*	2.80	1.859	1.888	1.858	16	1.853	28	1.852	40
*T. kodakarensis*	2.09	1.946	1.935	1.922	12	1.946	18	1.946	19
*M. jannaschii*	1.66	1.818	1.824	1.804	10	1.814	16	1.814	17
*M. genitalium*	0.58	1.818	1.841	1.812	4	1.816	4	1.816	4

Results regarding eleven complete genomes. Rates are in bits per base (bpb). The “DNA3” column contains the results provided by the technique of Manzini *et al.* using and order-3 fallback finite-context model. The “FCM-S” and “FCM-M” columns contain, respectively, the results provided by the single finite-context models and by the multiple competing finite-context models. The “XM50” and “XM200” columns show the results obtained with the XM algorithm, using 50 and 200 experts. Computation times, in seconds, are also included.

The probabilities associated to the finite-context models were estimated using (2), with 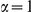 (corresponding to Laplace's estimator) for model orders 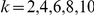 and with 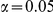 for model orders 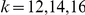. As explained in the previous section, when 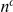 is large, the estimator converges to the maximum likelihood estimator, meaning that the value of 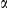 is virtually irrelevant when 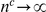. This is what happens for small-order models, because, due to the reduced number of contexts, on average the total number of events that occur associated to each context, i.e., 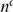, quickly attains a sufficiently high value for rendering 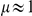 in (4). However, when 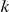 is large, then the number of conditioning states, 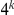, is very high. This implies that statistics have to be estimated using only a few observations (small values of 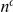), which is the case where the value of 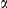 might play an important role.

In fact, during our study, we have found out experimentally that, using the combination of multiple finite-context models, the probability estimates calculated for the higher order models lead to significantly better results when smaller values 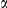 are used. We have performed a number of experiments and reached the conclusion that picking 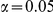 would provide, globally, good results. Other values similar to this one would also produce good results, meaning that the performance of the estimator is robust with respect to small variations of 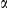.

The results presented in Table 3 show a clear distinction between organisms with small genomes and organisms with large ones. For small-sized genomes, the modeling ability of the multiple finite-context models is basically the same as the more sophisticated modeling approach provided by the XM algorithm. This is observed in the case of the *A. nidulans*, *C. albicans*, *S. pombe*, *S. cerevisiae*, *E. coli*, *S. aureus*, *T. kodakarensis*, *M. jannaschii* and *M. genitalium* organisms, with some of them being slightly better compressed by the finite-context models. For the *H. sapiens* and *A. thaliana* species, the modeling capability of the finite-context models appears to be insufficient, because the XM approach is able to attain lower entropies (about 5% lower when using 200 experts).

This result agrees with the known strong repetitive characteristic of the DNA data of the higher organisms, a characteristic that is usually better modeled by the copy expert mechanism provided by XM. On the contrary, the species with small-sized genomes seem to be very well represented exclusively by finite-context models, without needing the help of the copy experts. This observation allow us to conclude that the DNA sequence data of these species can be represented by models that rely only on short-term knowledge of the past, i.e., sixteen bases or less as suggested by the experimental results that we have obtained. Moreover, even in the higher species, the capability of the Markov-only approach seems to be quite significant, since it is able to represent, at least, about 95% of the information of the genome.

**Figure 2:**
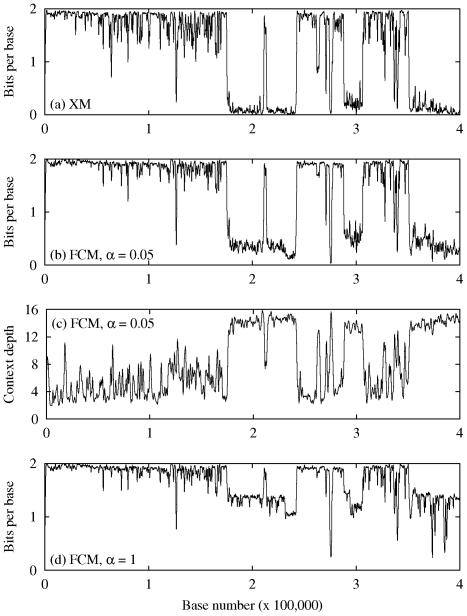
Example of information sequences for the first 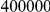 well-defined bases of human chromosome 1. (a) Information sequence generated by the XM method; (b) Information sequence generated by the multiple competing finite-context models, using 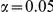 for the high-order models (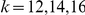) and 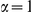 for the remainder models; (c) Variation of the depth of the context-model along the sequence, for the same setup as in (b); (d) The effect of parameter 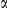. In this case, we show the information sequence generated by the multiple competing finite-context models with 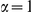 for all the models.

For better understanding how the two approaches behave locally, i.e., with and without the copy expert mechanism, Fig. 2 presents the information sequences regarding the first 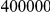 well-defined bases (i.e., ignoring the “N” cases) of the human chromosome number one. The (a), (b) and (d) plots represent the instantaneous number of bits required by each of the two modeling approaches for representing the DNA bases. Consequently, smaller values indicate that the DNA bases in that particular region of the DNA sequence were “easier” to represent (i.e., they required less bits) than other bases for which the values of the plot are higher. Note that, for facilitating the visualization of the curves, the data were low-pass filtered.

As can be seen, the curves displayed in Fig. 2(a) and (b) are reasonably similar. These plots exhibit valleys of varying depth mixed with a kind of plateau regions, clearly showing the different complexities that we referred along the paper and that motivated the adoption of the multiple competing finite-context models. The plateau-type regions reveal DNA segments that are difficult to represent, in the sense that they require more bits than average. These regions are typically encoded by the low-order finite context models. On the contrary, the valleys indicate DNA regions easier to represent, and, therefore, requiring less bits per DNA base. These parts of the DNA sequences are usually better handled by the high-order finite-context models or by the repetition-seeking mechanisms of the compression methods that incorporate this paradigm.

Still making use of the analytical power provided by the information sequences, Fig. 2(d) shows how important the value of 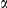 is in the probability estimator formula for a good performance of the high-order models. As can be seen in the figure, when using 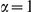 for all model depths the majority of the valleys is much less deep than when using 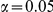 for the high-order models (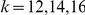), showing that the representation of the low complexity regions is strongly affected by this parameter.

Finally, in Fig. 2(c) we display the plot of the variation of the context depth along the sequence when processed with the same parameters as those used to produce the graphic shown in Fig. 2(b). It can be observed that, generally, deeper context models are chosen when the entropy is lower. Nevertheless, this is not always the case, and, therefore, these kind of plots may provide additional information about the structure of the DNA sequence.

### Conclusions

We have provided the first comprehensive investigation of the extent to which Markov models explain complete genomes. To explore the potential of Markov models as completely as possible, we have used a model that includes several competing Markov models of different orders. The model adapts to the statistical characteristics of the DNA sequences, which vary widely across the sequence, depending on the nature of the data (consider coding and non-coding DNA segments, for instance). The adaptation reflects itself on the selection of Markov models of different orders for different DNA segments.

We have noted that this approach is effective when complemented with the following ideas, that we have come to regard as essential: (a) careful programming, based on hash tables, to cope with the memory demands posed by the Markov models with longer context depth and the inherent sparsity of their associated contexts (b) probability estimates adequate to the wide range of context depths used (c) inverted repeat handling.

To measure the fit of the model at a certain position we adopted the negative logarithm of the probability estimate at that position. This standard measure yields information profiles of the sequences, which are of independent interest, and reveal instantaneous innovation along the sequences (that is, segments through which the sequence behaves in a more random and unpredictable way, as opposed to segments in which the behaviour is more predictable). The average of the measure over the entire sequence reduces to the average number of bits per base to describe the sequence, and works as a global performance measure.

A comparison of the results obtained with our multiple Markov model and state-of-the-art compression models reveals that the Markov-only description is able to explain genomes almost as well or even better. This is surprising for the following reasons. Our method is not intended to be a complete compression method – it does not attempt to explore long-range correlations and it does not take advantage of the presence of segments that are repeated (exactly or approximately) across the sequences. Furthermore, it consists only of Markov models, which are inherently short-range or local. Compression methods do take advantage of local correlations (and commonly resort to Markov models for that purpose) but also employ techniques such as copy experts, that are able to efficiently represent repetitions found along the sequence (potentially at unbounded distances). The fact that the degree of local dependence present in DNA sequences allows representations that compete with advanced compression methods is unexpected. The sequences for which our method gave better performance than state-of-the-art compressors (generally speaking, the shortest sequences) must include those for which short-range dependencies out-weight long-range dependencies. In other words, those that are less rich in exact and approximate repeats.

To conclude, our work provides evidence that complete DNA data sequences can be reasonably well described by statistical models that are inherently local, provided that inverted repeats are accounted for and that the probability estimates are taylored to the wide range of context depths used. Since the search for better data compression methods is closely related to the problem of finding better data models, this work contributes to an improved understanding of the laws that govern the DNA data.

## References

[1] Grumbach S, Tahi F (1993). Compression of DNA sequences..

[2] Rivals E, Delahaye JP, Dauchet M, Delgrange O (1996). A guaranteed compression scheme for repetitive DNA sequences..

[3] Loewenstern D, Yianilos PN (1997). Significantly lower entropy estimates for natural DNA sequences..

[4] Chen X, Kwong S, Li M (2001). A compression algorithm for DNA sequences.. IEEE Engineering in Medicine and Biology Magazine.

[5] Tabus I, Korodi G, Rissanen J (2003). DNA sequence compression using the normalized maximum likelihood model for discrete regression..

[6] Manzini G, Rastero M (2004). A simple and fast DNA compressor.. Software—Practice and Experience.

[7] Korodi G, Tabus I (2005). An efficient normalized maximum likelihood algorithm for DNA sequence compression.. ACM Trans on Information Systems.

[8] Behzadi B, Le Fessant F (2005). DNA compression challenge revisited.. Combinatorial Pattern Matching: Proc. of CPM-2005.

[9] Korodi G, Tabus I (2007). Normalized maximum likelihood model of order-1 for the compression of DNA sequences..

[10] Cao MD, Dix TI, Allison L, Mears C (2007). A simple statistical algorithm for biological sequence compression..

[11] Giancarlo R, Scaturro D, Utro F (2009). Textual data compression in computational biology: a synopsis.. Bioinformatics.

[12] Ziv J, Lempel A (1977). A universal algorithm for sequential data compression.. IEEE Trans on Information Theory.

[13] Borodovsky MY, Sprizhitsky YA, Golovanov EI, Aleksandrov AA (1986). Statistical patterns in primary structures of the functional regions of the genome in *Escherichia coli* : I. Frequency characteristics.. Molecular Biology.

[14] Borodovsky MY, Sprizhitsky YA, Golovanov EI, Aleksandrov AA (1986). Statistical patterns in primary structures of the functional regions of the genome in *Escherichia coli* : II. Nonuniform Markov models.. Molecular Biology.

[15] Tavaré S, Song B (1989). Codon preference and primary sequence structure in protein-coding regions.. Bulletin of Mathematical Biology.

[16] Borodovsky MY, McIninch J (1993). GENMARK: Parallel gene recognition for both DNA strands.. Computers & Chemistry.

[17] Burge CB, Karlin S (1998). Finding the genes in genomic DNA.. Current Opinion in Structural Biology.

[18] Salzberg SL, Pertea M, Delcher AL, Gardner MJ, Tettelin H (1999). Interpolated Markov models for eukaryotic gene finding.. Genomics.

[19] Stanke M, Waack S (2003). Gene prediction with a hidden Markov model and a new intron submodel.. Bioinformatics.

[20] Delcher AL, Bratke KA, Powers EC, Salzberg SL (2007). Identifying bacterial genes and endosymbiont DNA with Glimmer.. Bioinformatics.

[21] Zhu W, Lomsadze A, Borodovsky M (2010). *Ab initio* gene identification in metagenomic sequences.. Nucleic Acids Research.

[22] Rho M, Tang H, Ye Y (2010). FragGeneScan: predicting genes in short and error-prone reads.. Nucleic Acids Research.

[23] Robelin D, Richard H, Prum B (2003). SIC: a tool to detect short inverted segments in a biological sequence.. Nucleic Acids Research.

[24] Richard H, Nuel G (2003). SPA: simple web tool to assess statistical significance of DNA patterns.. Nucleic Acids Research.

[25] Liu Z, Chen D, Chen X (2007). CpG island identification with higher order and variable order Markov models..

[26] Rissanen J (1978). Modeling by shortest data description.. Automatica.

[27] Allison L, Edgoose T, Dix TI (1998). Compression of strings with approximate repeats..

[28] Dix TI, Powell DR, Allison L, Bernal J, Jaeger S (2007). Comparative analysis of long DNA sequences by per element information content using different contexts.. BMC Bioinformatics.

[29] Ferragina P, Giancarlo R, Greco V, Manzini G, Valiente G (2007). Compression-based classification of biological sequences and structures via the universal similarity metric: experimental assessment.. BMC Bioinformatics.

[30] Cao MD, Dix TI, Allison L (2010). A genome alignment algorithm based on compression.. BMC Bioinformatics.

[31] Allison L, Yee CN (1990). Minimum message length encoding and the comparison of macromolecules.. Bulletin of Mathematical Biology.

[32] Salamon P, Konopka AK (1992). A maximum entropy principle for the distribution of local complexity in naturally occurring nucleotide sequences.. Computers & Chemistry.

[33] Milosavljević A, Jurka J (1993). Discovering simple DNA sequences by the algorithmic significance method.. Computer Applications in the Biosciences.

[34] Bell TC, Cleary JG, Witten IH (1990). Text compression.. Prentice Hall.

[35] Salomon D (2007). Data compression - The complete reference..

[36] Sayood K (2006). Introduction to data compression..

[37] Pinho AJ, Neves AJR, Ferreira PJSG (2008). Inverted-repeats-aware finite-context models for DNA coding..

[38] Bayes T (1763). An essay towards solving a problem in the doctrine of chances.. Philosophical Transactions of the Royal Society of London.

[39] Laplace PS (1774). Mémoire sur la probabilité des causes par les événements..

[40] Venn J (1888). The logic of chance.

[41] Hardy GF (1889). Letter..

[42] Whittaker ET (1920). On some disputed questions of probability.. Trans of the Faculty of Actuaries.

[43] Johnson WE (1932). Probability: the deductive and inductive problems.. Mind.

[44] Zabell SL (1982). W. E. Johnson's “sufficientness” postulate.. The Annals of Statistics.

[45] Zabell SL (1989). The rule of succession.. Erkenntnis.

[46] Brown M, Hughey R, Krogh A, Mian IS, Sjölander K (1993). Using Dirichlet mixture priors to derive hidden Markov models for protein families..

[47] Sjölander K, Karplus K, Brown M, Hughey R, Krogh A (1996). Dirichlet mixtures: a method for improved detection of weak but significant protein sequence homology.. Bioinformatics.

